# Early prediction model of brain death in out-of-hospital cardiac arrest patients: a single-center retrospective and internal validation analysis

**DOI:** 10.1186/s12873-022-00734-1

**Published:** 2022-11-04

**Authors:** Yuki Itagaki, Mineji Hayakawa, Kunihiko Maekawa, Akira Kodate, Koyo Moriki, Yuki Takahashi, Hisako Sageshima

**Affiliations:** 1 Department of Surgery, Kushiro City General Hospital, 1-12, Shunko-Dai, Kushiro City, Hokkaido 085-0822 Japan; 2 Department of Emergency Medicine, Hokkaido University Hospital, Sapporo, Hokkaido Japan; 3 Emergency and Critical Care Centre, Sapporo City General Hospital, Hokkaido Sapporo, Japan; 4 Department of Emergency Medicine, Hakodate Municipal Hospital, Hakodate, Hokkaido Japan

**Keywords:** Brain death, Out-of-hospital cardiac arrest, Cardiopulmonary resuscitation, Organ donation, Prediction model

## Abstract

**Background:**

A shortage of donor organs amid high demand for transplantable organs is a worldwide problem, and an increase in organ donation would be welcomed by the global healthcare system. Patients with brain death (BD) are potential organ donors, and early prediction of patients with BD may facilitate the process of organ procurement. Therefore, we developed a model for the early prediction of BD in patients who survived the initial phase of out-of-hospital cardiac arrest (OHCA).

**Methods:**

We retrospectively analyzed data of patients aged < 80 years who experienced OHCA with a return of spontaneous circulation (ROSC) and were admitted to our hospital between 2006 and 2018. We categorized patients into either a non-BD or BD group. Demographic and laboratory data on ED admission were used for stepwise logistic regression analysis. Prediction scores of BD after OHCA were based on β-coefficients of prognostic factors identified in the multivariable logistic model.

**Results:**

Overall, 419 OHCA patients with ROSC were admitted to our hospital during the study period. Seventy-seven patients showed BD (18.3%). Age and etiology of OHCA were significantly different between the groups. Logistic regression analysis confirmed that age, low-flow time, pH, and etiology were independent predictors of BD. The area under the receiver operating characteristic curve for this model was 0.831 (95% confidence interval, 0.786–0.876).

**Conclusions:**

We developed and internally validated a new prediction model for BD after OHCA, which could aid in the early identification of potential organ donors for early donor organ procurement.

## Background

A shortage of donor organs amid high demand for transplantable organs is a worldwide problem [1–5], and an increase in organ donation would be welcomed by the global healthcare system [6]. Patients with brain death (BD) are potential organ donors. Thus, early prediction of patients with BD may facilitate the process of organ procurement. Few studies have established predictive models for detecting BD [7]. In a spontaneous intracerebral haemorrhage, poor corneal reflexes and swirl signs on an initial computed tomography scan are good predictors of BD (sensitivity: 0.89; specificity: 0.68) [7].

Despite recent progress in resuscitative and critical care medicine, mortality after out-of-hospital cardiac arrest (OHCA) remains high. Approximately 300,000 cardiac arrests occur in the United States, and 100,000 occur in Japan annually [8]. Since the brain is the most susceptible organ to hypoxia and inadequate organ perfusion [9], some OHCA patients, unfortunately, develop total loss of brain function (i.e., BD) in the first several days after the return of spontaneous circulation (ROSC) [10]. A futility score (NULL-PLEASE) predicting poor outcomes in OHCA patients was developed [11] and externally validated by a group in the United Kingdom [12]. However, its predictive ability for fatal outcomes was not strong enough [11, 12]. The bispectral index during extracorporeal cardiopulmonary resuscitation (E-CPR) for cardiac arrest is also useful in predicting BD [13]. A bispectral index below 30 is associated with a sensitivity of 96% and a specificity of 82% for detecting BD occurrence in patients treated with E-CPR.

Because OHCA is associated with complicated patient characteristics, situations, and environments, detailed information gathered by the emergency department (ED) on admission can be used to obtain a clear idea of the patient’s health status and situation. Given that these variables are immediately available at the time of admission to ED, certain clinical factors contributing to BD can be detected. However, only a few prediction models have been developed for detecting BD in OHCA patients. Therefore, we aimed to identify significant predictive variables to develop and validate a discriminative model for the early prediction of BD in patients who survived the initial phase of an OHCA.

## Methods

### Patients

We performed a retrospective analysis using data from electronic medical records of patients who experienced OHCA with ROSC and admitted to Hokkaido University Hospital, Hokkaido, Japan between 2006 and 2018.

Patients aged under 80 years were eligible for this study, as those aged over 80 years are unlikely to be donor candidates. The records of eligible patients were searched to collect laboratory test results at admission to ED, along with data on clinical characteristics, treatments, and patient outcomes.

### Definition

BD was defined as a state of deep coma, electrocerebral inactivity, loss of brainstem reflexes, dilation (≥ 4 mm) and fixing of the pupils, and apnea. A diagnosis of BD was made based on the attending physician’s judgement. We categorised patients into two groups: non-BD and BD. Low-flow time was defined as the time from the initiation of chest compressions to ROSC and was included as a continuous variable. Bystander CPR was defined as the initiation of CPR by a witness of the cardiopulmonary arrest (Utstein style).

### Statistical analyses

The Mann–Whitney U and chi-square tests were used for comparisons between the two groups. We inputted the following demographic and laboratory data regarding ED admission into the stepwise logistic regression model: age, sex, the presence of a bystander who witnessed cardiac arrest, initial rhythm, presence of a shockable rhythm, serum lactate level, low-flow time, pH of arterial blood, and the presence or absence of end-stage renal failure requiring dialysis. All these variables were investigated at admission to ED. Demographic and characteristic data were collected by the Emergency Medical Service, and the serum test results were automatically recorded in electrical medical records. The corresponding author extracted the data from the medical records of Hokkaido University Hospital. Each continuous variables were changed into categorial variables by classification and regression tree models to determine the cut off value of continuous variables such as pH, lactate, low-flow time. Then, we put these variables into stepwise logistic regression analysis to confirm the variables that were both readily available at admission and predictive of BD. The prediction scores for BD after OHCA were based on the β-coefficients of prognostic factors identified from a multivariable logistic model. The calculated β-coefficients were rounded to the nearest integer to reflect the weight of each variable. A binary logarithmic function model was developed according to the score. We estimated the predictability of the score using the area under the receiver operating characteristic curve (AUROC). Youden’s index was used to determine the cutoff value of the OHCA-BD score for predicting BD. Internal validation was performed by bootstrapping using a bias-corrected and accelerated method based on 1000 bootstrapped samples, and we assessed the discrimination by determining the AUROC for each original dataset and the 1000 computed samples. Optimism was estimated by calculating the AUROC using 1000 bootstrapped resamples of the dataset and calculating the differences between the AUROCs of all bootstrapped resamples and the original dataset. The Hosmer–Lemeshow test was performed to confirm score calibration. All statistical analyses were performed with R software (version 3.5.1). All tests were two-tailed, and *p*-values of 0.05 or less were considered statistically significant.

## Results

A total of 419 OHCA patients with ROSC were admitted to our hospital during the study period. Their demographic and clinical characteristics are presented in Table 1. The overall BD rate was 18.3% (*n* = 77). The values for age, lactate level, and low-flow time were significantly higher in the BD group than in the non-BD group. The male-to-female ratio and proportions of witnessed status and defibrillation were lower in BD patients than in non-BD patients. OHCAs due to cerebrovascular disease were much more likely to lead to BD. Furthermore, pH was significantly lower in the BD group. Although lactate levels, low-flow time, pH, and sex were not significantly different between groups, age and OHCA aetiology were significantly different.

**Table 1:** Demographic and clinical characteristics of the patients after out-of-hospital cardiac arrest

Parameters	All patients (*n* = 419)					
	Non-BD			BD *n* = 77	*P* value	
	All *n* = 342	Survived *n* = 198	Death *n* = 144		All non-BD vs. BD	Non-BD Death vs. BD
Age (IQR)	66 (56, 74)	64 (55, 74)	67 (58, 75)	56 (42, 64)	< 0.001	< 0.001
Male (%)	232(67.8)	136 (68.7)	96 (66.7)	41 (53.2)	0.017	0.059
Etiology (%)						
Internal						
Cardiac	215 (62.9)	137 (69.2)	78 (54.2)	24 (31.2)	< 0.001	0.004
Respiratory	25 (7.3)	13 (6.6)	12 (8.8)	9 (11.7)		
Cerebrovascular	20 (5.8)	7 (3.5)	13 (9.0)	18 (23.4)		
External	58 (17.0)	31 (15.7)	27 (18.8)	20 (26.0)		
Unknown	24 (7.0)	10 (5.1)	14 (9.7)	6 (7.8)		
Witness (%)	227 (66.4)	156 (78.8)	73 (50.7)	40 (51.9)	0.025	0.778
Bystander CPR (%)	195 (57.0)	104 (52.5)	91(63.2)	41(53.2)	0.611	0.154
Initial rhythm upon EMS arrival (%)						
ROSC	11 (3.2)	9 (4.5)	2 (1.4)	2 (2.6)	0.036	0.399
VF/pulseless VT	137 (40.1)	98 (49.5)	39 (27.1)	18 (23.4)		
PEA	64 (18.7)	38 (19.2)	26 (18.1)	21 (27.3)		
Asystole	119 (34.8)	48 (24.2)	71 (49.3)	35 (45.5)		
Unknown	11 (3.2)	5 (2.5)	9 (6.2)	1 (1.3)		
Defibrillation (%)	162 (47.4)	110 (47.5)	52 (36.1)	21 (27.3)	0.001	0.230
Low-flow time, min (IQR)	25.5 (15.0, 40.8)	20.0 (11.25, 34.8)	31.5 (21.0, 43.0)	35.0 (25.0, 46.0)	< 0.001	0.192
BGA on arrival at ED						
pH (IQR)	7.08 (6.89, 7.30)	7.21 (7.00, 7.33)	6.97 (6.80, 7.14)	6.91 (6.82, 7.03)	< 0.001	0.167
Base deficit, mmol/L (IQR)	13.55 (6.72, 21.08)	10.2 (4.4, 16.5)	18.8 (12.10, 23.7)	20.10 (14.10, 23.50)	< 0.001	0.526
Lactate, mmol/L (IQR)	9.80 (6.23, 13.17)	7.9 (4.4, 11.6)	11.6 (9.1, 14.9)	12.70 (9.20, 14.60)	0.001	0.906
End-stage renal failure on dialysis (%)	14 (4.1)	5 (2.5)	9 (6.2)	1 (1.3)	0.324	0.170

*IQR* Interquartile range, *BD* Brain death, *CPR* Cardiopulmonary resuscitation, *ROSC* Return of spontaneous circulation, *VF* Ventricular fibrillation, *VT* Ventricular tachycardia, *PEA* Pulseless electrical inactivity, *BGA* Blood gas analysis, *BE* Base deficit, *ED* Emergency departmen

Classification and regression tree models were applied to convert continuous variables to categorical variables. The thresholds for each continuous value were as follows: age (< 43 years, 43–67 years, > 67 years), pH (< 6.62, 6.62–7.06, ≥ 7.06), low-flow time (< 20 min, ≥ 20 min), and lactate level (< 8.75 mmol/L, 8.75–12.65 mmol/L, ≥ 12.65 mmol/L). After categorizing the continuous variables, stepwise logistic regression analysis was performed, which confirmed that age, low-flow time, pH, and etiology were independent predictors of BD (Table 2). The developed prediction score (OHCA-BD score) is presented in Table 3. The corrected coefficient was defined as the sum of several variables based on the rounded nearest integer to reflect the weight of each variable.

**Table 2:** Results of the stepwise multivariate logistic regression analysis

Coefficients	β coefficient	Odds ratio	95% CI	*P*-value
Intercept	-2.7717	0.06	(0.02—0.19)	< 0.01
Age				
< 43 years	0			reference
43–67 years	-1.024	0.36	(0.17—0.78)	< 0.01
≥ 67 years	-2.4262	0.09	(0.04—0.22)	< 0.01
Etiology				
Internal				
Cardiac	0			reference
Respiratory	1.5243	4.59	(1.71—12.3)	< 0.01
Cerebrovascular	2.1272	8.39	(3.47—20.3)	< 0.01
External	1.2052	3.34	(1.57—7.1)	< 0.01
Unknown	0.7364	2.09	(0.71—6.19)	0.18
Low-flow time				
< 20 min	0			reference
≥ 20 min	1.7976	6.03	(2.18—16.7)	< 0.01
< 6.62	0			reference
pH				
6.62–7.06	0.6964	2.01	(0.74—5.44)	0.17
≥ 7.06	-0.1774	0.84	(0.27—2.6)	0.76

Low-flow time means the time from the initiation of chest compressions to the return of spontaneous circulation

**Table 3:** Categorical classification of each variable for brain death

Variables	β coefficient	corrected coeffcient
Intercept	-2.7717	
Age		
< 43 years	0	14
43–67 years	-1.024	6
≥ 67 years	-2.4262	0
Etiology		
Internal		
Cardiac	0	0
Respiratory	1.5243	9
Cerebrovascular	2.1272	12
External	1.2052	7
Unknown	0.7364	4
Low-flow time		
< 20 min	0	0
≥ 20 min	1.7976	10
pH		
< 6.62	0	1
6.62—7.06	0.6964	5
≥ 7.06	-0.1774	0

Low-flow time means the time from the initation of chest compression to return of spontaneous circulation

**Fig. 1:**
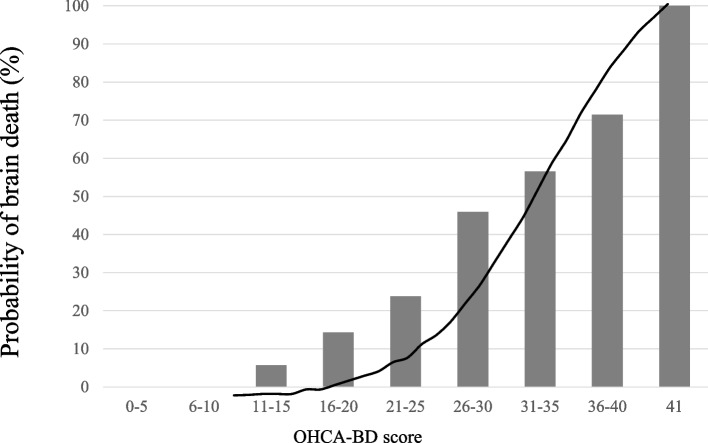
Relationship between the rate of brain death and OHCA-BD score. The line graph shows the predicted probability of brain death according to the OHCA-BD score; the bar graph shows the actual rate of brain death according to the OHCA-BD score.. OHCA-BD, out-of-hospital cardiac arrest-brain death

**Fig. 2:**
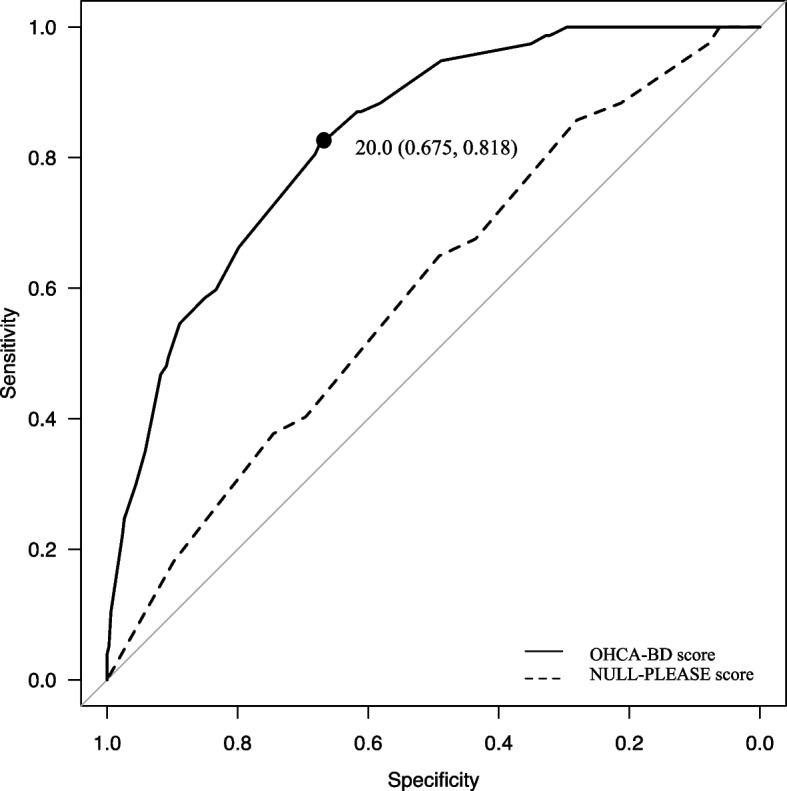
Areas under the ROC curves of OHCA-BD and NULL-PLEASE scores for predicting brain death. The cutoff point of the OHCA-BD score to predict brain death was 20 points (sensitivity 67.5% and specificity 81.8%). Solid line, ROC curve of OHCA-BD score; dotted line, ROC curve of NULL-PLEASE score. ROC, receiver operating characteristic; OHCA-BD, out-of-hospital cardiac arrest-brain death

Consequently, we created a linearised logarithmic function model using this score. In Fig. 1, the predictive probability of BD according to this score is shown by a line graph, and the actual rate of BD is shown by a bar graph. The AUROC for this model was 0.831 (95% confidence interval [CI], 0.786–0.876) (Fig. 2). The cut-off point was 20/41 (sensitivity 67.5%; specificity 81.8%).

**Fig. 3:**
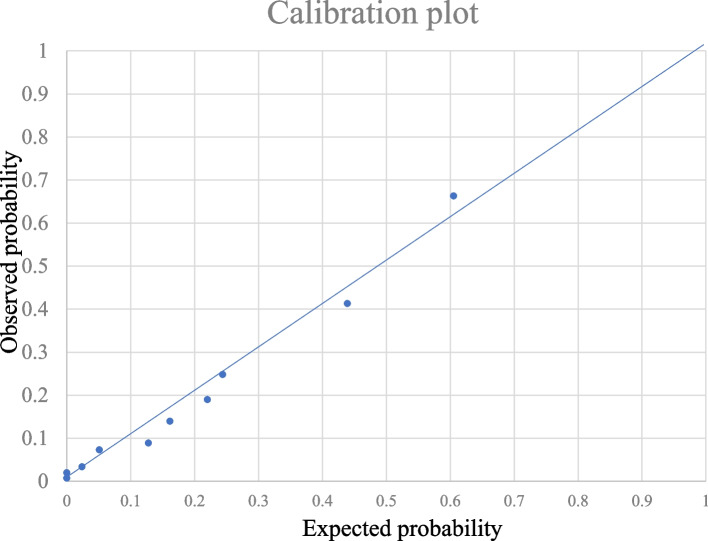
Hosmer–Lemeshow Calibration plot. This calibration plot revealed a good predictive accuracy of the scoring model

Internal validation of the score using bootstrapping yielded an average optimism of 0.0015. The Hosmer–Lemeshow chi-squared value was 4.3674 (degrees of freedom = 6), and the non-significant *p*-value of 0.6271 and the calibration plot (Fig. 3) indicated a good model fit.

We also calculated the AUROC of the NULL-PLEASE score to determine its ability to predict BD. However, its prediction ability for BD was not high enough (AUROC 0.596; 95% CI 0.53–0.66) and was significantly different compared with the OHCA-BD score (*p* < 0.01) (Fig. 2).

## Discussion

We developed a model to predict BD based on a relatively large population of OHCA patients admitted to our hospital between 2006 and 2018. The prognostic variables for the early prediction of BD were age, low-flow time, pH, and aetiology. This scoring model was evaluated by internal validation and was shown to be a good predictor for BD. To increase the rate of organ donation and decide on the clinical course after OHCA, this scoring model may be a useful tool in the early setting after OHCA.

According to demographic data, there are specific variables in patients with BD. For example, younger age is an independent predictor of BD. This may be because the brain of a younger person occupies less space in the cranium than those of older individuals. Furthermore, haematomas due to head injury and multiple hemispheric lesions with oedema due to cardiac arrest contribute to brain herniation, resulting in BD due to compression of the brainstem. In addition, older patients tend to die due to circulatory collapse, which avoids progression toward BD. Younger individuals without any background disease compared with older individuals would increase the possibility of ROSC.

OHCA due to cerebrovascular disease correlated most strongly with BD in terms of aetiology. This may be because of direct compression of the brain stem by the corresponding lesion such as haematoma or oedema mentioned above. However, other parameters of tissue hypoperfusion, including lactate, pH, and low-flow time, were indicators of poor outcomes but not specific indicators of BD. For example, excessive long low-flow time and low pH were not statistically correlated with BD. We speculated that moderate hypoperfusion status would be required to detect BD.

Since the brain is the most susceptible organ to hypoxia and inadequate perfusion, OHCA contributes enormously to BD. The rate of organ donation from patients with BD following OHCA was reported to be 5.4% in a single-centre study from the United Kingdom [14], which is not high. Certainly, coordination of organ donation through the decedent’s family is essential for organ donation from patients with BD because the refusal rate for organ donation remains high. However, in many cases of OHCA, circulation is refractory, and the patient dies without confirmation of BD or fails to be diagnosed with BD. Furthermore, withdrawing life-sustaining therapy because of perceived poor neurological prognosis is the primary cause of hospital-related death after OHCA [15, 16]. It should be noted that our hospital does not make early decisions to withdraw mechanical, ventilated, or organ-perfusion support. Therefore, among patients with a poor neurological prognosis in our retrospective cohort, we could distinguish between patients who developed BD and those who died without developing BD, similar to the study by Galbois et al. [7]. We have the same concepts and rules, which leads to increased length of stay due to which patients might develop BD. Therefore, our intensive care practice did not introduce bias by preventing BD development.

Ahmad et al. introduced the NULL-PLEASE score, which predicts poor neurological outcomes in OHCA patients [11]. However, the NULL-PLEASE score could not predict the development of BD in the present study. The poor neurological prognosis predicted by the NULL-PLEASE score model includes severe disability, vegetative state, and death. As BD is only a limited part of the poor neurological outcome, the NULL-PLEASE model may not predict BD precisely. Therefore, to predict progression to BD, a specific prediction scoring model is needed.

In another predicting model of BD [17], the prediction variables were female gender, non-shockable rhythm, cardiac cause of OHCA, neurological cause of OHCA, natremia after 24 h, and vasoactive drug at admission and at 24 h. This study used data from two multi-centre randomised controlled trials (RCTs), with a total population of 675 patients, of whom 84 were patients with BD (12%), and the AUROC was approximately 0.8. Compared with this study, our study, with an AUROC of 0.831, was not so inferior to that of another prognostic study for BD. Furthermore, this study had two serious selection biases; one RCT only included non-shockable OHCA patients who have less than 30 min of no-flow time, and the decision to withdraw care was at the discretion of each treating team [18], and another RCT included only cardiac-origin OHCA patients [19]. Although the present study is a single-centre retrospective study, our patients have less selection bias because of the specific aetiology of OHCA, continuous care without any withdrawal, and non-limited inclusion criteria for OHCA other than those under 80 years of age.

Organ donation is a stressful and difficult end-of-life decision causing anxiety, depression, and decreased quality of life among family members of the deceased donor [20]. The family needs to fully understand that BD means the death of their loved one and that we need consent from the family for organ donation [21]. This process is also ethically and emotionally challenging for the physicians involved [22]. We may be able to explain in advance the family for the possible outcome and decisions such as end-of-life care or organ donation by objective assessment using a predictive score.

Taking these concerns into consideration, the early detection of BD is merely one step aimed toward respecting the patient’s and family’s decisions. Furthermore, the medical status of patients with BD should be suitable for organ donation even if the family consents. Thus, organ donation is a tough achievement only after overcoming major social and medical problems, and this novel scoring model developed in this study would help ED or ICU physicians to predict BD.

Limitations of this study include the single-centre design, possible selection bias, and confounding by unknown or unmeasured variables. We might also have a bias in determining the treatment for OHCA patients. Although bootstrapping is a strong tool for statistical internal validation, it cannot avoid overlapping of patients selected for each dataset. Furthermore, we did not perform external validation of the prediction score in this study. As the sensitivity and specificity are not perfect, this scoring model alone cannot determine the clinical outcome. As this is a single-centre retrospective study, we should consider whether this scoring system is applicable for universal implementation. Further prospective observational studies are needed for external validation of our results.

## Conclusion

In conclusion, a new predictive model for BD after OHCA was developed and internally validated. By using this scoring model, patients at high risk of BD after OHCA may be identified at early stages.

## Data Availability

The datasets generated during and/or analyzed during the current study are available from the corresponding author on reasonable request.

## Abbreviations

OHCA: Out-of-hospital cardiac arrest
ROSC: Return of spontaneous circulation
E-CPR: Extracorporeal cardiopulmonary resuscitation
ED: Emergency department
BD: Brain death
AUROC: Area under the receiver operating characteristic curve
RCT: Randomised controlled trial
